# Why translational research matters: proceedings of the third international symposium on acute lung injury translational research (INSPIRES III)

**DOI:** 10.1186/s40635-019-0230-9

**Published:** 2019-07-25

**Authors:** Nicole P. Juffermans, Marcus Schultz, Lieuwe D. Bos, Oscar Penuelas, John Laffey, Jose A. Lorente

**Affiliations:** 1 Amsterdam University Medical Center, Amsterdam, the Netherlands; 2 Servicio de Medicina Intensiva, Hospital Universitario de Getafe, Madrid, Spain; 3 Spain CIBER de Enfermedades Respiratorias, Universidad Europea, Madrid, Spain; 4 Department of Intensive Care, National University of Ireland, Regenerative Medicine Institute (REMEDI) at CÚRAM Centre for Research in Medical Devices, Galway, Ireland NUI, Galway, Ireland

**Keywords:** Translational research, ARDS, Acute lung injury, Models

## Abstract

Current treatment of acute respiratory distress syndrome (ARDS) in critically ill patients is limited to supportive measures including mechanical ventilation. It is our view that effective therapies for ARDS can only be found through experimental and translational science that seeks to better understand the mechanisms of injury and identify therapeutic approaches, using pre-clinical models of acute lung injury that closely mimic the clinical syndrome of ARDS. This editorial gives examples of ways in which translational science contributes to the development of more specific measures against ARDS in the critically ill. In order to improve focus on this essential research as well as to enhance collaborative research efforts, a Translational Biology Group was founded within the European Society of Intensive Care Medicine. INSPIRES is an international symposium on translational research in lung injury as well as in other areas of critical illness, intended to serve as a platform for the translational biology working group. This issue of ICMx is dedicated to the proceedings of INSPIRES III.

## Background

Current treatment of acute respiratory distress syndrome (ARDS) in critically ill patients is limited to supportive measures including mechanical ventilation. Extensive translational research using acute lung injury models has identified potential harmful effects of ventilation, resulting in the identification of effective protective measures, such as the use of lower tidal volume, use of restrictive fluids, use of lower pressures, and ventilation in the prone position [1–4]. Despite these advancements, the mortality of ARDS remains unacceptably high [5]. Currently, specific therapies for ARDS are lacking. Most reviews covering the topic of ARDS end with the hope that we will find such specific therapies. It is our view that such therapies can only be found through experimental and translational science that seeks to better understand the mechanisms of injury and identify therapeutic approaches, using pre-clinical models of acute lung injury that closely mimic the clinical syndrome of ARDS. This issue of ICMx is dedicated to the proceedings of INSPIRES III, an international symposium on acute pulmonary injury translational research, organized for the third time, and this year held in Amsterdam, The Netherlands.

From a broad perspective, there are several ways in which translational research may contribute to the development of more specific measures against ARDS in the critically ill. These include improved understanding of the pathophysiology of the injured lung, testing of potential therapeutic strategies in models that closely mimic the complexities of ARDS, stratification of patients in future randomized clinical trials, and improvement of understanding of the outcome of such trials.

## Improving our understanding of the pathophysiology of lung injury

Translational research augments the understanding of the pathogenesis and pathophysiology of lung injury in the critically ill. As examples, the rational use of PEEP or prone positioning or low tidal volume is all advances guided by a better understanding of pathophysiology. In addition to management options, understanding of the pathophysiology of lung injury has also improved diagnostic options. Examples are exhaled breath analysis, e.g., to distinguish infectious from non-infectious lung injury [6].

## Testing of potential therapeutic strategies in ARDS models

Advancements in insight into the genetics and biology of ARDS can aid in the testing of therapeutic strategies in ARDS. An example may be the recognition that ARDS is characterized by a local pro-coagulant pulmonary response [7]. Therapeutic strategies that have been tested are numerous, including nebulized activated protein C, antithrombin, or heparin, which all have been found to attenuate lung injury in lung injury models [8–10]. Another example is the use of knock-out animals in lung injury models, in order to determine whether specific pathways play a role that could be targeted. A relevant example may be plasminogen activator inhibitor-1 (PAI-1). In a hyperoxic lung injury model, animals that were deficient of PAI-1 were resistant to the development of lung injury [11]. PAI-1 is heterogeneously expressed among the population, and the outcome has been linked to homozygous carriage of the 4G allele of the PAI-1 gene with increased levels of PAI-1 [12]. Consequently, lung injury models may be useful to determine whether the genetic background of ARDS patients should be taken into account when designing an interventional trial with an agent targeting a specific pathway.

## Stratification of patients for inclusion in clinical trials

Numerous interventions that were shown to be beneficial in preclinical settings did not improve outcomes in subsequent clinical trials. In ARDS, examples are the use of simvastatin and inhaled beta-agonists [13, 14]. Another example is corticosteroids, which have shown contrasting responses in different ARDS patient populations [15]. An explanation may be that even when intervention seems rational from a pathophysiologic point of view, recipients may respond differently because the underlying “biology” (i.e., mechanisms of injury and/or host response) is different.

In ARDS, patients have historically been classified according to the severity of hypoxemia. The PaO_2_:FiO_2_ ratio is part of the original ARDS definition as well as of the current Berlin definition and is used to classify patients into mild, moderate, and severe ARDS [16, 17]. The inclusion of the more severe ARDS patients in clinical trials that tested “physiologic” interventions has led to positive outcome findings. These include trials of neuromuscular blockade and prone positioning [2, 18], suggesting that classification according to hypoxemia may be useful. This may be due to an improved understanding of the physiology of ARDS. Alternatively, the efficacy of ventilatory interventions such as prone positioning may depend more on the severity of lung injury and less on ‘ARDS specific’ processes.

Besides physiology or disease severity, it may also be possible to further categorize ARDS patients who may benefit from interventions, based on a more “biologic” profile. Re-analysis of two large interventional trials suggest that two specific subphenotypes may exist, one of which is characterized by more severe inflammation, shock, and metabolic acidosis, corroborating with significantly worse clinical outcomes [19]. Interestingly, the PaO_2_:FiO_2_ ratio did not distinguish these subphenotypes. In line with this, another study found that ARDS patients can be clustered based on specific combinations of biomarkers, resulting in either an “uninflamed” or a “reactive” biological phenotype, which were associated with mortality. Again, differences in mortality between the biological phenotypes were independent of the clinical classification into mild, moderate, and severe ARDS [20]. These phenotypes had a differential response to simvastatin therapy, with a favorable response in the hyperactive inflammatory subtype, but not in the hypo inflammatory subtype [21]. The existence of different subphenotypes in patients with the clinical diagnosis of ARDS is also suggested by the results of a large postmortem study, in which a different clinical profile (more severe organ dysfunction, worse respiratory system mechanics) was identified in patients with diffuse alveolar damage (DAD) as the lung histological finding as compared with patients without DAD [22]. Relevant to this finding is that in trial testing corticosteroids, ARDS patients with low fibroproliferative activity as reflected by lower baseline lung levels of procollagen peptide type III, responded favorably to steroids, whereas patients with high fibroproliferative activity did not [15].

The challenge, however, is to find biomarkers which can identify these and other subphenotypes at the bedside, prior to randomizing patients into clinical trials.

Taken together, translational research in ARDS should not make simplistic extrapolations from preclinical models to all patients with ARDS. If we want to translate findings from models studying biology into treatments for patients, we should acknowledge the need to target potentially effective treatments for ARDS to patients with the specific biologic characteristics targeted by that therapeutic approach.

## A better understanding of results from clinical trials

Thus far, we have discussed translational research from the classical viewpoint: as a tool to close the gap between knowledge produced at the lab bench and its use at the patient’s bedside. However, translational research may also help us the other way around, in improving our understanding of results from clinical trials. An example may be the open lung approach. Mechanical ventilation can induce or worsen lung injury, referred to as ventilator-induced lung injury (VILI). Mechanisms of VILI include alveolar overdistension as well as the repetitive opening and closing of alveoli during breathing. As a consequence, use of lower tidal volume combined with higher levels of positive end-expiratory pressure (PEEP) to prevent overdistension and avoid repetitive collapse and reopening of alveolar units have been suggested as a protective ventilatory strategy, together with recruitment maneuvers prior to setting of PEEP, referred to as the open lung approach. However, clinical trials on the use of the open lung approach that have used a clinically relevant outcome measure show conflicting results, suggesting both improved as well as worsened outcome [23–25]. These conflicting results challenged the concept that aggressive strategies to reverse atelectasis during respiratory failure are beneficial. In line with this, experimental studies suggest a reduced inflammatory host response in lung areas with atelectasis as compared to non-dependent lung areas [26]. Possibly, this is explained by a delivery of higher tidal volumes to the non-atelectatic lung regions without promoting excessive opening and closing of alveolar units [27]. Another example is the use of neuromuscular blocking agents, which decreases mortality when used in the early stages of ARDS [18]. The mechanism by which this occurs is however unclear. Models have improved our understanding by showing that paralysis abolishes patient-ventilator dyssynchrony, which decreases oxygen consumption and reduces the inflammatory response associated with ARDS. Thereby, translational research may also be useful to fill in gaps from the bedside to the bench.

## INSPIRES: a platform to promote basic science in acute respiratory failure

In recognition of the important role of experimental and translational science in improving care for patients with respiratory failure, the first INSPIRES symposium was held in Madrid in 2016. It was rated as a great success and resulted in research collaborations as well as in sharing of laboratory skills and techniques through exchange programs of PhD students.

A similar effort on stimulating translational research in Intensive Care Medicine is the founding of the Translational Biology Group (TBG). This working group is related to the ESICM Acute Respiratory Failure Working Group but aims to have a broad focus on experimental and translational science.

Specific objectives of TBG and INSPIRES are to identify the current research questions in (acute respiratory failure and other critical illnesses that can be answered with experimental and translational science to improve collaborations and exchange between research groups and to provide a platform for the presentation of basic research. Currently, speakers on the symposia of intensive care mainly represent senior researchers who give “state-of-the-art” overview lectures. INSPIRES aims to take a slightly different approach, encouraging young investigators to present their data.

This special issue contains the proceedings of INSPIRES III. Chapters provide background on the research questions and related data that were presented at the meeting, such as the use of biomarkers and ultrasound in diagnosing and monitoring of lung injury, how to optimize mechanical ventilation, and experimental data involving novel therapeutic interventions. It is our hopes that efforts such as INSPIRES will contribute to the improvement of care for the critically ill.
